# A Three-Year Observational Study of the Prevalence of Left Ventricular Diastolic Dysfunction in Asymptomatic Individuals With Cardiovascular Risk Factors

**DOI:** 10.7759/cureus.100575

**Published:** 2026-01-01

**Authors:** Milan Mehta, Nileshkumar Khanpara

**Affiliations:** 1 Cardiology, Sardar Patel Hospital and Heart Institute, Bharuch, IND; 2 General Medicine, Om Hospital and ICU, Surat, IND

**Keywords:** asymptomatic, diabetes mellitus, diastolic dysfunction, echocardiography, hypertension, obesity, screening

## Abstract

Background: Left ventricular diastolic dysfunction (LVDD) precedes heart failure and may remain undetected in asymptomatic individuals. Early identification is particularly relevant in populations with cardiometabolic risk factors, where subclinical LVDD could refine risk stratification and guide preventive strategies.

Objective: To determine the prevalence of clinically significant LVDD (grade II-III) among asymptomatic adults aged 51-70 years and to compare its burden across major cardiovascular risk groups.

Methods: This retrospective cross‑sectional study analyzed 10,434 transthoracic echocardiograms performed over three years (between 1^st ^October 2022 and 30^th ^September 2025) at a tertiary cardiac center. A total of 5,482 asymptomatic individuals aged 51-70 years were included after exclusion of symptomatic patients and patients with structural heart disease, ejection fraction <50%, arrhythmias, and grade I LVDD. Participants were categorized into healthy controls, isolated hypertension, isolated diabetes mellitus, combined hypertension and diabetes mellitus, and severe obesity (BMI >40 kg/m²). Diastolic function was assessed using guideline‑based Doppler echocardiographic criteria, and only grade II-III LVDD was classified as clinically significant.

Results: Clinically significant LVDD was present in 102 healthy controls (3.7%, 95% CI: 3.0-4.5). Prevalence was higher in hypertension (13.1%, 95% CI: 11.4-15.0), diabetes mellitus (10.9%, 95% CI: 9.0-13.1), and combined hypertension plus diabetes (29.1%, 95% CI: 24.4-33.8). The highest prevalence occurred in severe obesity (53.5%, 95% CI: 34.1-72.2). LVDD prevalence increased stepwise across escalating cardiometabolic risk profiles.

Conclusion: Clinically significant LVDD is uncommon in asymptomatic individuals without risk factors but substantially more prevalent in those with hypertension, diabetes, or severe obesity. The graded increase across risk categories underscores the cardiometabolic contribution to diastolic dysfunction. While routine screening of all asymptomatic individuals is not supported, targeted evaluation of high‑risk groups warrants further prospective investigation.

## Introduction

Heart failure with preserved ejection fraction (HFpEF) accounts for nearly half of all heart failure cases and is associated with substantial morbidity and mortality [1,2]. Left ventricular diastolic dysfunction (LVDD), a central abnormality in HFpEF, may remain clinically silent for years before symptoms develop [3,4]. During this asymptomatic phase, progressive impairment of myocardial relaxation and compliance can gradually culminate in overt HFpEF [5,6]. Because early LVDD is typically unaccompanied by clinical manifestations, it is often unrecognized until advanced stages, when therapeutic options are limited and outcomes less favorable [5,6].

Hypertension, diabetes mellitus, and obesity are well-established contributors to LVDD, yet their relative and combined impact on clinically significant diastolic dysfunction in asymptomatic individuals remains incompletely defined [7-10]. Cardiometabolic risk factors frequently coexist and may exert cumulative effects, accelerating subclinical myocardial remodeling. Despite these associations, current guidelines do not recommend routine echocardiographic screening in asymptomatic individuals, including those with multiple cardiovascular risk factors [2,10]. This conservative position reflects limited population-level evidence and ongoing uncertainty regarding the clinical relevance of subclinical LVDD detected in the absence of symptoms [11-14].

Importantly, the present study focuses on asymptomatic individuals undergoing clinically indicated, non-emergent echocardiography rather than population-based screening, a distinction that is relevant when interpreting prevalence estimates and generalizability.

To address this gap, we conducted a three-year observational study to evaluate the prevalence of grade II and grade III LVDD across distinct cardiovascular risk categories in asymptomatic adults aged 51-70 years. Our aim was to quantify the burden of clinically significant LVDD within each risk group and to assess how prevalence varies across escalating cardiometabolic profiles, thereby providing population-level insights into the earliest detectable stages of HFpEF pathogenesis [7-9,12,13].

The study was restricted to individuals aged 51-70 years to balance several considerations. This age range excludes younger adults in whom LVDD is uncommon and diastolic parameters remain relatively stable, while also excluding individuals above 70 years in whom age-related physiological changes can confound interpretation of diastolic function independent of cardiometabolic risk factors. This middle-aged to early elderly population represents the critical window during which subclinical LVDD typically emerges in the presence of cardiovascular risk factors, and is the demographic group most likely to benefit from early detection and targeted preventive interventions before progression to symptomatic heart failure [5,6,14,15].

## Materials and methods

Study design and population

This observational cross-sectional study analyzed data from transthoracic echocardiography (TTE) examinations performed at a tertiary cardiac care center over a three-year period (1 October 2022 to 30 September 2025). During this period, a total of 10,434 TTE studies were performed. From these, 5,482 asymptomatic individuals were identified and enrolled based on predefined eligibility criteria. These individuals underwent echocardiography for non-emergent clinical indications, including routine health screening and preoperative cardiovascular evaluation.

Eligible participants were required to meet predefined inclusion criteria, including age between 51 and 70 years, complete absence of cardiac symptoms such as dyspnea, chest pain, palpitations, syncope, or exercise intolerance, and availability of a comprehensive transthoracic echocardiographic examination with adequate image quality for detailed structural and Doppler assessment. Participants were also required to have accessible medical records documenting cardiovascular risk factor status.

Individuals were excluded if they had any cardiac symptoms at presentation, known structural heart disease (including valvular disease, cardiomyopathy, or congenital heart disease), prior myocardial infarction or coronary revascularization, atrial fibrillation or other clinically significant arrhythmias, left ventricular ejection fraction below 50%, or inadequate echocardiographic windows or incomplete Doppler assessment. Grade I diastolic dysfunction was prespecified as an exclusion criterion, as its prognostic significance in asymptomatic individuals remains uncertain and it is commonly regarded as an age-related physiological finding rather than clinically significant disease [3,11-14].

Participants were categorized according to cardiovascular risk factor profiles. Hypertension was defined by a documented diagnosis in medical records or current use of antihypertensive therapy. Diabetes mellitus was defined by a documented diagnosis or current treatment with oral hypoglycemic agents or insulin. Combined hypertension and diabetes mellitus was defined by the presence of both conditions. Body mass index was recorded for all participants and analyzed as a continuous variable; for descriptive phenotypic classification, severe obesity was defined as a body mass index greater than 40 kg/m² in the absence of hypertension and diabetes mellitus, representing an isolated obesity phenotype [7-10]. Healthy controls were defined as asymptomatic individuals without any of the above cardiovascular risk factors.

To minimize confounding related to age, an established independent determinant of LVDD [5,6], the analysis was restricted to individuals aged 51-70 years. This approach excluded age extremes while focusing on a population at meaningful risk for cardiovascular disease and HFpEF [1,2], thereby enhancing internal validity and facilitating clearer assessment of associations between cardiometabolic risk factors and LVDD.

All echocardiographic examinations were performed using a GE Vivid E9 ultrasound system equipped with XDclear probe technology (GE Healthcare, Chicago, IL). Studies were acquired by experienced cardiac sonographers and interpreted by board-certified cardiologists with expertise in echocardiography. Standard imaging included two-dimensional and M-mode acquisitions in parasternal and apical views, supplemented by a comprehensive Doppler assessment. Doppler evaluation included pulsed-wave Doppler of mitral inflow at the leaflet tips, tissue Doppler imaging of the mitral annulus at septal (medial) and lateral sites, pulmonary venous flow assessment when feasible, and measurement of tricuspid regurgitation velocity for estimation of pulmonary artery systolic pressure.

Left ventricular diastolic function was assessed in accordance with contemporary guidelines and recommended multiparametric criteria [4,15]. Mitral inflow analysis included measurement of early (E) and late (A) diastolic velocities and calculation of the E/A ratio. Tissue Doppler imaging was used to measure early diastolic mitral annular velocity (e′) at both septal and lateral sites, with calculation of average e′ velocity. The E/e′ ratio was derived as an estimate of left ventricular filling pressures. When mitral inflow patterns suggested possible pseudonormalization, the Valsalva maneuver was performed as an adjunctive measure to aid diastolic function grading.

Only grade II (pseudonormal) and grade III (restrictive) diastolic dysfunction were classified as clinically significant and included in the analysis [4,15]. Grade II diastolic dysfunction was identified based on a combination of mitral inflow parameters, reduced e′ velocity, elevated E/e′ ratio, and supportive findings such as changes during the Valsalva maneuver, consistent with guideline recommendations. Grade III diastolic dysfunction was defined by a restrictive filling pattern characterized by a markedly elevated E/A ratio, reduced e′ velocity, elevated E/e′ ratio, and shortened deceleration time. Grade I diastolic dysfunction was excluded a priori due to its uncertain prognostic significance in asymptomatic populations [3,11-14]. In these definitions, E represents early diastolic mitral inflow velocity, A represents late diastolic mitral inflow velocity, and e′ represents early diastolic mitral annular tissue velocity. All echocardiographic examinations were interpreted by board-certified cardiologists experienced in diastolic function assessment and graded according to standardized, guideline-based criteria to minimize interobserver variability.

Statistical analyses

Statistical analyses were performed using Python (Python Software Foundation, Wilmington, DE) and R (R Foundation for Statistical Computing, Vienna, Austria) statistical environments. Continuous variables were expressed as mean ± standard deviation, and categorical variables as frequencies and percentages. Prevalence estimates of clinically significant LVDD (grade II-III) were calculated for each cardiovascular risk category together with corresponding 95% confidence intervals.

Comparisons of LVDD prevalence across the predefined risk groups (healthy controls, isolated hypertension, isolated diabetes mellitus, combined hypertension and diabetes mellitus, and severe obesity) were conducted using the chi-square test for independence. To evaluate the presence of systematic age-related trends in LVDD prevalence within individual risk groups, an ordinal Cochran-Armitage trend test was applied across the 20-year age span (51-70 years).

To quantify the association between age and LVDD, grouped binomial logistic regression models were fitted separately for hypertension, diabetes mellitus, and combined hypertension and diabetes mellitus cohorts. Odds ratios (ORs) for LVDD were reported per one-year increase in age, along with 95% confidence intervals and model fit indices.

Given the non-linear distribution of LVDD across BMI categories, two complementary modeling strategies were implemented. First, a piecewise (segmented) logistic regression model with a prespecified breakpoint at BMI 27 was used to estimate differential slopes below and above the threshold. Second, restricted cubic spline (RCS) modeling was applied to characterize continuous non-linear associations and to visualize inflection points across the full BMI range.

Model adequacy and comparative performance were assessed using the Akaike information criterion (AIC), Bayesian information criterion (BIC), and Nagelkerke pseudo-R². All statistical tests were two-sided, and a p-value <0.05 was considered statistically significant.

Ethical considerations

This study was approved by the Institutional Ethics Committee. Given the retrospective nature of the study and the use of de-identified clinical data obtained as part of routine patient care, the requirement for individual informed consent was waived by the ethics committee. All data were anonymized prior to analysis, and the study was conducted in accordance with the principles of the Declaration of Helsinki.

## Results

Study population characteristics

Over the three-year study period, 10,434 transthoracic echocardiographic examinations were performed at our center. After application of predefined inclusion and exclusion criteria, 5,482 asymptomatic individuals were included in the final analysis. Of these, 2,747 participants were asymptomatic and without any of the studied cardiovascular risk factors, while 2,735 participants were asymptomatic but had at least one cardiovascular risk factor.

Within the risk-factor cohort, isolated hypertension constituted the largest subgroup, followed by isolated diabetes mellitus. Smaller proportions of participants had combined hypertension and diabetes mellitus, while severe obesity as an isolated phenotype represented a relatively small subset of the study population. This distribution reflects the predominance of hypertension and diabetes as common cardiometabolic risk factors among asymptomatic individuals undergoing echocardiographic evaluation.

Table 1 summarizes the baseline demographic and echocardiographic characteristics across the risk categories. The mean age of the overall study population was 59.9 years and was comparable across all subgroups, indicating that age was well balanced and unlikely to account for observed differences in LVDD prevalence.

**Table 1:** Baseline population characteristics. Baseline demographic and echocardiographic parameters stratified by cardiovascular risk category. Values are mean ± SD or n (%). LVDD: left ventricular diastolic dysfunction (grade II or III); BMI: body mass index; HTN: hypertension; DM: diabetes mellitus; TR: tricuspid regurgitation.

Variable	Healthy controls (n = 2,747)	Hypertension (n = 1,424)	Diabetes mellitus (n = 922)	HTN + DM (n = 361)	Severe obesity (n = 28)	Total (n = 5,482)
Age, years (mean ± SD)	59.7 ± 5.5	60.1 ± 5.6	59.8 ± 5.7	60.2 ± 5.4	59.5 ± 5.8	59.9 ± 5.6
Male sex, n (%)	1,420 (51.7)	780 (54.8)	480 (52.0)	190 (52.6)	10 (35.7)	2,880 (52.5)
Female sex, n (%)	1,327 (48.3)	644 (45.2)	442 (48.0)	171 (47.4)	18 (64.3)	2,602 (47.5)
BMI, kg/m² (mean ± SD)	25.1 ± 3.2	27.8 ± 4.1	27.2 ± 3.9	28.5 ± 4.5	42.8 ± 2.1	26.9 ± 4.6
Mitral E/A ratio (mean ± SD)	0.92 ± 0.18	0.88 ± 0.20	0.87 ± 0.19	0.82 ± 0.22	0.79 ± 0.25	0.89 ± 0.20
Septal e′ velocity (cm/s)	8.9 ± 1.6	7.6 ± 1.5	7.8 ± 1.4	6.9 ± 1.3	6.2 ± 1.2	8.2 ± 1.5
Lateral e′ velocity (cm/s)	11.2 ± 1.8	9.8 ± 1.7	10.0 ± 1.6	8.9 ± 1.5	8.0 ± 1.4	10.4 ± 1.7
Average e′ velocity (cm/s)	10.1 ± 1.7	8.7 ± 1.6	8.9 ± 1.5	7.9 ± 1.4	7.1 ± 1.3	9.3 ± 1.6
E/e′ ratio (mean ± SD)	9.8 ± 2.1	12.4 ± 2.5	12.1 ± 2.4	14.8 ± 2.9	16.2 ± 3.1	11.5 ± 2.6
Deceleration time (ms)	210 ± 25	195 ± 28	198 ± 27	180 ± 30	170 ± 32	200 ± 28
TR velocity (m/s)	2.4 ± 0.3	2.6 ± 0.4	2.5 ± 0.4	2.7 ± 0.5	2.8 ± 0.5	2.5 ± 0.4
LVDD grade II, n (%)	95 (3.5)	170 (11.9)	90 (9.8)	92 (25.5)	12 (42.8)	459 (8.4)
LVDD grade III, n (%)	7 (0.2)	17 (1.2)	11 (1.1)	13 (3.6)	3 (10.7)	51 (0.9)

The prevalence of clinically significant LVDD varied across cardiovascular risk categories (Table 1). Compared with asymptomatic individuals without risk factors, both hypertension and diabetes mellitus were associated with higher LVDD prevalence. Individuals with coexisting hypertension and diabetes mellitus demonstrated a substantially greater prevalence of LVDD than those with either condition alone, consistent with an incremental association between cumulative cardiometabolic burden and LVDD risk.

Table 2 illustrates the findings from the piecewise logistic regression analysis examining the relationship between body mass index (BMI) and clinically significant LVDD. A breakpoint at BMI 27 was identified, below which LVDD prevalence remained stable, and above which each one‑unit increase in BMI was associated with a 24% higher odds of LVDD. This inflection highlights the overweight‑to‑obesity transition as a critical threshold for diastolic dysfunction risk.

**Table 2:** Piecewise logistic regression of BMI and LVDD prevalence (breakpoint = 27). Piecewise logistic regression demonstrating the relationship between BMI and clinically significant LVDD. A breakpoint at BMI 27 was identified. Below this threshold, LVDD prevalence remained stable, while above it, each one‑unit increase in BMI was associated with a 24% higher odds of LVDD. This inflection highlights the overweight‑to‑obesity transition as a critical threshold for diastolic dysfunction risk. BMI: body mass index; LVDD: left ventricular diastolic dysfunction; SE: standard error; OR: odds ratio; 95% CI: 95% confidence interval.

Predictor	β (Coefficient)	SE	OR	95% CI	p‑value
Intercept	–6.687	5.065	—	—	0.187
BMI ≤27 slope	0.152	0.197	1.16	0.79 – 1.71	0.440
BMI >27 slope	0.217	0.056	1.24	1.11 – 1.39	

**Figure 1:**
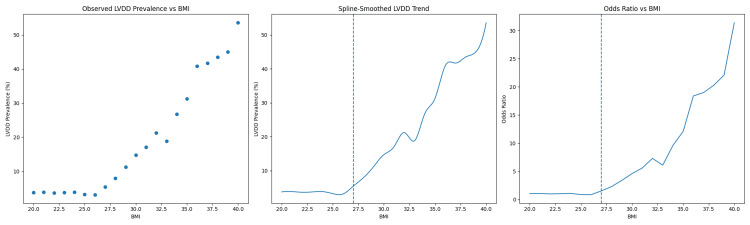
Association between body mass index and clinically significant left ventricular diastolic dysfunction. The left panel shows the observed prevalence of grade II–III LVDD (%) across increasing body mass index (BMI) categories. The middle panel depicts a spline-smoothed trend illustrating the non-linear association between BMI and LVDD prevalence. The right panel presents the adjusted odds ratio for LVDD across BMI values. The vertical dashed line indicates a BMI of 27.5 kg/m², representing the conventional Asian cut-off for overweight. Overall, LVDD prevalence and odds increase progressively with higher BMI, particularly beyond this threshold. BMI: body mass index; LVDD: left ventricular diastolic dysfunction.

Figure 1 illustrates the relationship between body mass index (BMI) and clinically significant LVDD. The left panel shows the observed prevalence of grade II-III LVDD across increasing BMI values. The middle panel presents a spline-smoothed curve demonstrating a non-linear association between BMI and LVDD prevalence. The right panel depicts the corresponding odds ratios for LVDD across BMI values. Together, these panels demonstrate a progressive increase in LVDD prevalence and odds with higher BMI, particularly beyond the mid-to-upper BMI range.

A markedly higher prevalence of LVDD was also observed among individuals with severe obesity (BMI >40 kg/m²). Although the number of participants in this subgroup was limited, the observed increase suggests a potential association between severe obesity and preclinical LVDD that may warrant further investigation. Overall, these findings demonstrate a graded increase in LVDD prevalence across escalating cardiometabolic risk profiles in an asymptomatic population.

**Figure 2:**
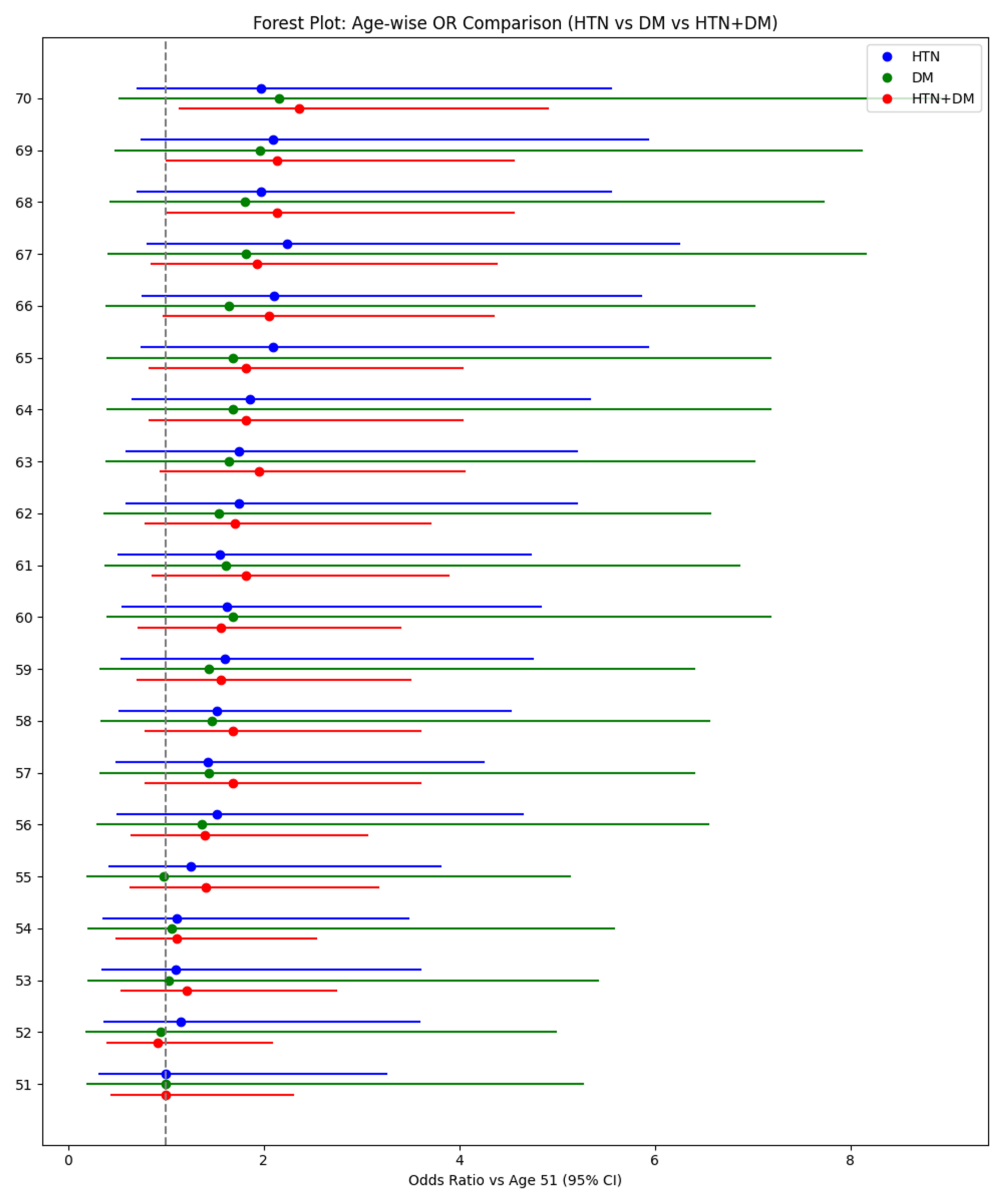
Forest plot: Age-wise odds ratio comparison for LVDD (hypertension vs. diabetes vs. hypertension + diabetes). This forest plot illustrates age-specific odds ratios (ORs) for left ventricular diastolic dysfunction (LVDD) comparing hypertensive (blue), diabetic (green), and combined hypertension-plus-diabetes (red) groups, referenced against age 51. Each point represents the OR for a given age, and horizontal lines denote the corresponding 95% confidence intervals (CIs). The vertical dashed line at OR = 1 represents the null value. Across increasing age, a progressive rise in ORs is observed in all groups, with the highest ORs consistently seen in the combined HTN+DM group, followed by isolated hypertension and isolated diabetes, indicating a synergistic effect of combined cardiometabolic risk factors on LVDD. HTN: hypertension; DM: diabetes mellitus.

Figure 2 presents age-specific odds ratios for clinically significant LVDD across cardiometabolic risk groups, referenced to age 51. Odds ratios increased progressively with advancing age in all groups, with the steepest rise observed in individuals with combined hypertension and diabetes. Hypertension alone showed consistently higher odds than diabetes, but both were exceeded by the dual‑risk group, underscoring the synergistic effect of coexisting cardiometabolic conditions. The forest plot highlights the cumulative impact of age and multiple risk factors on LVDD risk.

Interpretation of multivariable regression findings

Multivariable regression results are summarized in Table 3. The multivariable logistic regression models demonstrate that BMI and age are the strongest independent predictors of clinically significant LVDD after adjusting for sex, hypertension, and diabetes mellitus. In all three models, each 1 kg/m² increase in BMI was associated with a 7-8% higher odds of LVDD, with consistent statistical significance (p <0.001), underscoring the robust influence of excess adiposity on diastolic function. Hypertension and diabetes mellitus, although associated with higher crude prevalence, were not independently significant predictors in the fully adjusted model, suggesting that much of their effect overlaps with or is mediated through BMI-related mechanisms (Table 3). The significant interaction between BMI and hypertension in models B and C indicates a synergistic relationship, where the adverse effect of higher BMI on LVDD is amplified in individuals with coexisting hypertension. Age also remained a consistent predictor across models, with each additional year conferring approximately 5% higher odds of LVDD, reflecting the known age-related decline in diastolic function. Taken together, these findings highlight that increasing BMI and older age are principal contributors to subclinical LVDD, and that obesity-hypertension interplay substantially accelerates diastolic impairment even in asymptomatic individuals.

**Table 3:** Multivariable logistic regression models for clinically significant LVDD. Odds ratios (ORs) with 95% confidence intervals (CIs) derived from multivariable logistic regression models adjusted for age and sex. Model A includes main effects only. Model B includes an interaction term between BMI and hypertension. Model C includes interaction terms between BMI and hypertension and BMI and diabetes mellitus. BMI: body mass index; HTN: hypertension; DM: diabetes mellitus; LVDD: left ventricular diastolic dysfunction.

Variable	Model A: Main effects OR (95% CI)	p-value	Model B: BMI × HTN OR (95% CI)	p-value	Model C: BMI × HTN + BMI × DM OR (95% CI)	p-value
BMI (per 1 kg/m²)	1.08 (1.06–1.10)	<0.001	1.07 (1.05–1.09)	<0.001	1.07 (1.05–1.09)	<0.001
Hypertension	1.21 (0.96–1.53)	0.11	1.15 (0.90–1.47)	0.27	1.14 (0.89–1.46)	0.30
Diabetes mellitus	1.12 (0.88–1.44)	0.34	1.10 (0.86–1.42)	0.45	1.08 (0.83–1.40)	0.57
BMI × Hypertension	—	—	1.04 (1.01–1.07)	0.01	1.04 (1.01–1.07)	0.01
BMI × Diabetes mellitus	—	—	—	—	1.02 (0.99–1.05)	0.18
Age (per year)	1.05 (1.03–1.06)	<0.001	1.05 (1.03–1.06)	<0.001	1.05 (1.03–1.06)	<0.001
Male sex	1.18 (0.97–1.43)	0.09	1.17 (0.96–1.43)	0.12	1.16 (0.95–1.41)	0.14

## Discussion

This three-year observational study demonstrates that clinically significant LVDD is detectable in a substantial proportion of asymptomatic adults, particularly among those with cardiometabolic risk factors. Prevalence was lowest in healthy controls and increased progressively across hypertension, diabetes, combined hypertension and diabetes, and severe obesity. The markedly higher prevalence in individuals with concurrent hypertension and diabetes underscores the synergy of multiple risk factors on early myocardial impairment [16]. Although crude prevalence was higher in hypertension and diabetes, multivariable models indicated that BMI and age were stronger independent predictors, suggesting mediation through adiposity and age [17].

Previous population-based studies have reported diastolic dysfunction prevalence estimates ranging from 20% to 30% [11-14]. Direct comparisons are limited by methodological differences, including broader age ranges, variable diagnostic criteria, and frequent inclusion of grade I dysfunction. By restricting analysis to grade II-III LVDD, our study emphasizes clinically meaningful abnormalities more consistently associated with adverse outcomes [18]. This approach likely underestimates the overall burden of diastolic abnormalities but improves interpretability and provides a clearer epidemiologic benchmark.

Notably, BMI exhibited a non-linear relationship with LVDD. Both piecewise logistic regression and restricted cubic spline modeling demonstrated a stable, low prevalence of LVDD between a BMI of 21 and 26, followed by a clear inflection around BMI 27 and a steep rise thereafter, indicating that excess adiposity contributes meaningfully to diastolic dysfunction only after a critical threshold is crossed.

The observed gradient in LVDD prevalence across risk categories is consistent with established pathophysiological mechanisms. Hypertension is associated with chronic pressure overload and concentric remodeling, diabetes mellitus with myocardial stiffness through advanced glycation and microvascular disease [16], and severe obesity with increased hemodynamic demand, systemic inflammation, and neurohormonal activation. In individuals with combined hypertension and diabetes, the coexistence of these mechanisms may amplify LVDD risk, although causal inferences cannot be drawn from this cross-sectional design.

These findings highlight the heterogeneous distribution of LVDD risk across asymptomatic populations and raise important considerations for risk stratification. While current guidelines do not recommend routine echocardiographic screening in asymptomatic individuals [2,4,10], our results suggest that selected high-risk subgroups may harbor a clinically significant burden of LVDD. A risk-stratified screening approach could warrant further evaluation to determine whether early identification prompts more effective preventive strategies or improved outcomes.

Strengths of this study include restriction to adults aged 51-70 years to minimize age-related confounding [17], a large sample size of 5,482 individuals, and use of standardized echocardiographic protocols with guideline-directed multiparametric assessment. Limitations of this study include its retrospective, cross-sectional design, small subgroup sizes (particularly among individuals with severe obesity), lack of detailed data on the duration or control of cardiometabolic risk factors, potential interobserver variability in diastolic function assessment, and the single-center setting. In addition, although echocardiography was performed for non-emergent indications, the study population represents a clinically referred cohort, introducing potential referral bias and limiting the generalizability of the findings to the broader asymptomatic population.

Future research should focus on prospective longitudinal studies to define the natural history of subclinical LVDD [18], randomized trials to evaluate whether targeted identification improves outcomes, and integration of biomarkers and advanced imaging into risk stratification. These efforts may clarify whether early recognition of LVDD in high-risk asymptomatic individuals can inform preventive strategies and reduce progression to HFpEF.

## Conclusions

Clinically significant LVDD was identified in a notable proportion of asymptomatic adults with cardiometabolic risk factors, particularly those with multiple coexisting conditions. Prevalence increased stepwise across risk categories, highlighting higher observed rates of clinically significant LVDD across escalating cardiometabolic risk profiles. While routine echocardiographic screening in asymptomatic populations is not recommended, these findings indicate that targeted evaluation of selected high‑risk groups merits further investigation. Prospective studies are needed to determine whether early detection of LVDD, combined with intensified risk-factor management, is associated with improved outcomes and represents a cost-effective strategy.
